# A novel fully automatic design approach of a 3D printed face specific mask: Proof of concept

**DOI:** 10.1371/journal.pone.0243388

**Published:** 2020-12-03

**Authors:** Eman Shaheen, Robin Willaert, Isabel Miclotte, Ruxandra Coropciuc, Michel Bila, Constantinus Politis

**Affiliations:** 1 Department of Oral and Maxillofacial Surgery, University Hospitals Leuven, Leuven, Belgium; 2 Department of Imaging and Pathology, OMFS IMPATH Research Group, Faculty of Medicine, KU Leuven, Leuven, Belgium; Eberhard-Karls-Universitat Tubingen Medizinische Fakultat, GERMANY

## Abstract

The use of high quality facemasks is indispensable in the light of the current COVID pandemic. This study proposes a fully automatic technique to design a face specific mask. Through the use of stereophotogrammetry, computer-assisted design and three-dimensional (3D) printing, we describe a protocol for manufacturing facemasks perfectly adapted to the individual face characteristics. The face specific mask was compared to a universal design of facemask and different filter container’s designs were merged with the mask body. Subjective assessment of the face specific mask demonstrated tight closure at the nose, mouth and chin area, and permits the normal wearing of glasses. A screw-drive locking system is advised for easy assembly of the filter components. Automation of the process enables high volume production but still allows sufficient designer interaction to answer specific requirements. The suggested protocol can be used to provide more comfortable, effective and sustainable solution compared to a single use, standardized mask. Subsequent research on printing materials, sterilization technique and compliance with international regulations will facilitate the introduction of the face specific mask in clinical practice as well as for general use.

## Introduction

Personal protective equipment (PPE) and social distancing are the cornerstones for prevention of disease transmission of the Sars-CoV-2 virus (severe acute respiratory syndrome coronavirus 2). The viral particles are present in the upper respiratory tract for more than 2 weeks after symptom onset [1]. The virus is spreading predominantly through respiratory droplets (sneezing, coughing) or indirect transmission with contaminated surfaces [2]. All health care providers working nearby the face are at high risk, notably when they are exposed to aerosols or using rotating instruments, e.g. for dentist and oral-maxillofacial surgeons [1, 3].

As no vaccine is currently available, all human individuals and healthcare providers in particular, have to rely on the quality of PPE to protect their own health and prevent nosocomial spreading when close interaction cannot be avoided [4]. In the context of the current pandemic, the demand for high quality FFP2 (N95) and FFP3 (N99) masks, outstrips the worldwide supply, more than ever since lockdown exit strategies require population-wide wearing of face masks [5]. Furthermore, standard masks are not fitted to the individual facial anatomy and could provoke skin pressure ulcers when continuous wearing is obligatory [6]. These reasons necessitate exploring alternative solutions that are efficient, easy to produce and durable.

Computer assisted design and manufacturing (CAD-CAM) has become widely available during the last decades [7]. Open source databases, freeware and low-cost three-dimensional (3D) printers have boosted the possibilities to create a variety of products, including facemasks. However, the lack of personal modifications will prevent a close fit to the face of the person who wears it. An air-tight closure is essential to avoid leakage of contaminated air in or out the breathing zone and provide sufficient protection. If 3D printed masks are to be used as an alternative and safe solution for the regular masks, it is important that a tight fit and comfortable wearing can be achieved [8]. Additional prerequisites for a sustainable solution are the ability to reuse the masks and make the manufacturing process as straightforward as possible.

The aim of this study is to present a fully automatic approach to design a 3D face specific mask that provides optimal protection and comfort.

## Materials and methods

### Proposed design protocol

Ethical approval was obtained from the Ethical Review Board of the University Hospitals Leuven, Belgium (reference no: B322201316317), in compliance with the Helsinki Declaration. A test subject volunteered for this study and has given written informed consent (as outlined in PLOS consent form) to publish these case details. As this paper is describing the steps to an algorithm, a sample size of one is considered appropriate to this proof of concept paper. The proposed algorithm can be divided into 6 main steps as described in details in the following subsections.

**Fig 1:**
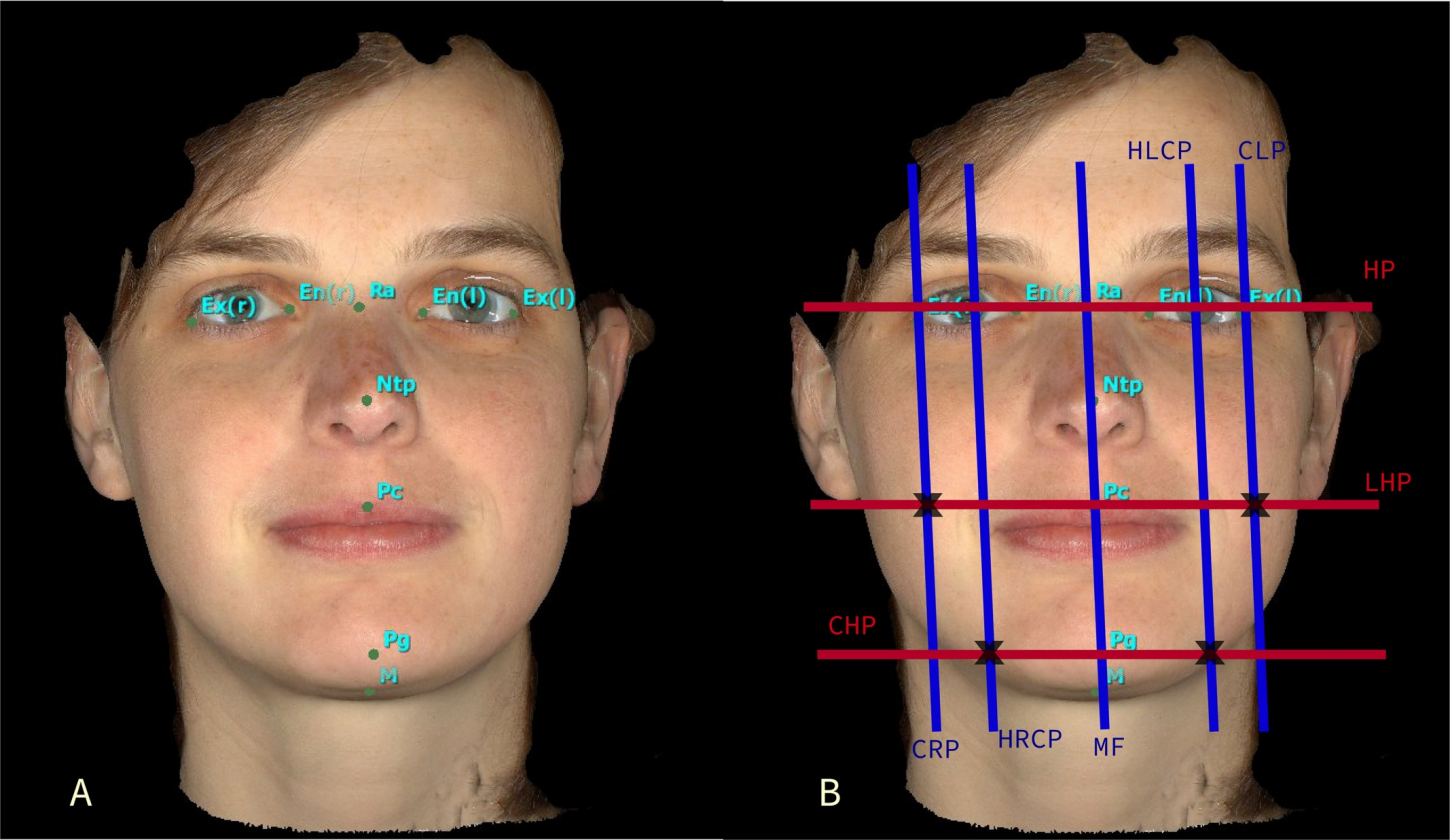
A. 3D image of the test subject with automatically annotated nine landmarks from VECTRA® Face Sculptor® software. B. The 3D image with the calculated planes represented as lines (red: HP, LHP, CHP; blue: MF, HLCP, CLP, HRCP, CRP) for simplicity showing their intersection (black **X**) to form the calculated control points.

**Step 1: Image acquisition and landmarks indication.** Three-dimensional stereophotogrammetric image of the subject was taken in a relaxed normal face expression using a handheld 3D Vectra® H1 (Canfield Scientific Inc., Parsippany, NJ, USA) camera after calibration of the device as described by Ayaz et al. [9]. After construction of the 3D image, the VECTRA® Face Sculptor® software places soft tissue landmarks automatically as shown in Fig 1A. The coordinates of a subset of these landmarks, i.e. nine landmarks, (Table 1 and Fig 1A) are exported for the next step along with the 3D image (F3D) that is exported in OBJ file format.

**Table 1:** Anatomical landmarks, calculated points and planes.

***Anatomical landmarks***
Lateral Canthus right: Ex(r)
Medial Canthus right: En(r)
Radix: Ra
Medial Canthus left: En(l)
Lateral Canthus left: Ex(l)
Nasal Tip: Ntp
Philtral Crest: Pc
Pogonion: Pg
Menton: M
***Calculated planes***
Midface (MF) is a plane through 3 points: Ra, Ntp, Pg
Vertical plane (VP) is a plane through Ex(l), Ex(r) and perpendicular to MF
Horizontal plane (HP) is a plane through Ra and perpendicular to MF and VP
Lip horizontal plane (LHP) is a plane through Pc and parallel to HP
Chin horizontal plane (CHP) is a plane through Pg and parallel to HP
Hallow of left cheek plane (HLCP) is a plane through Ex(l) and parallel to MF
Hallow of right cheek plane (HRCP) is a plane through Ex(r) and parallel to MF
Chin left plane (CLP) is a plane through midpoint between Ex(l) and En(l) and parallel to MF
Chin right plane (CRP) is a plane through midpoint between Ex(r) and En(r) and parallel to MF
***Calculated control points***
C1: Ra
C2: intersection point between HRCP, LHP and face surface
C3: intersection point between CRP, CHP and face surface
C4: M
C5: intersection point between CLP, CHP and face surface
C6: intersection point between HLCP, LHP and face surface

**Fig 2:**
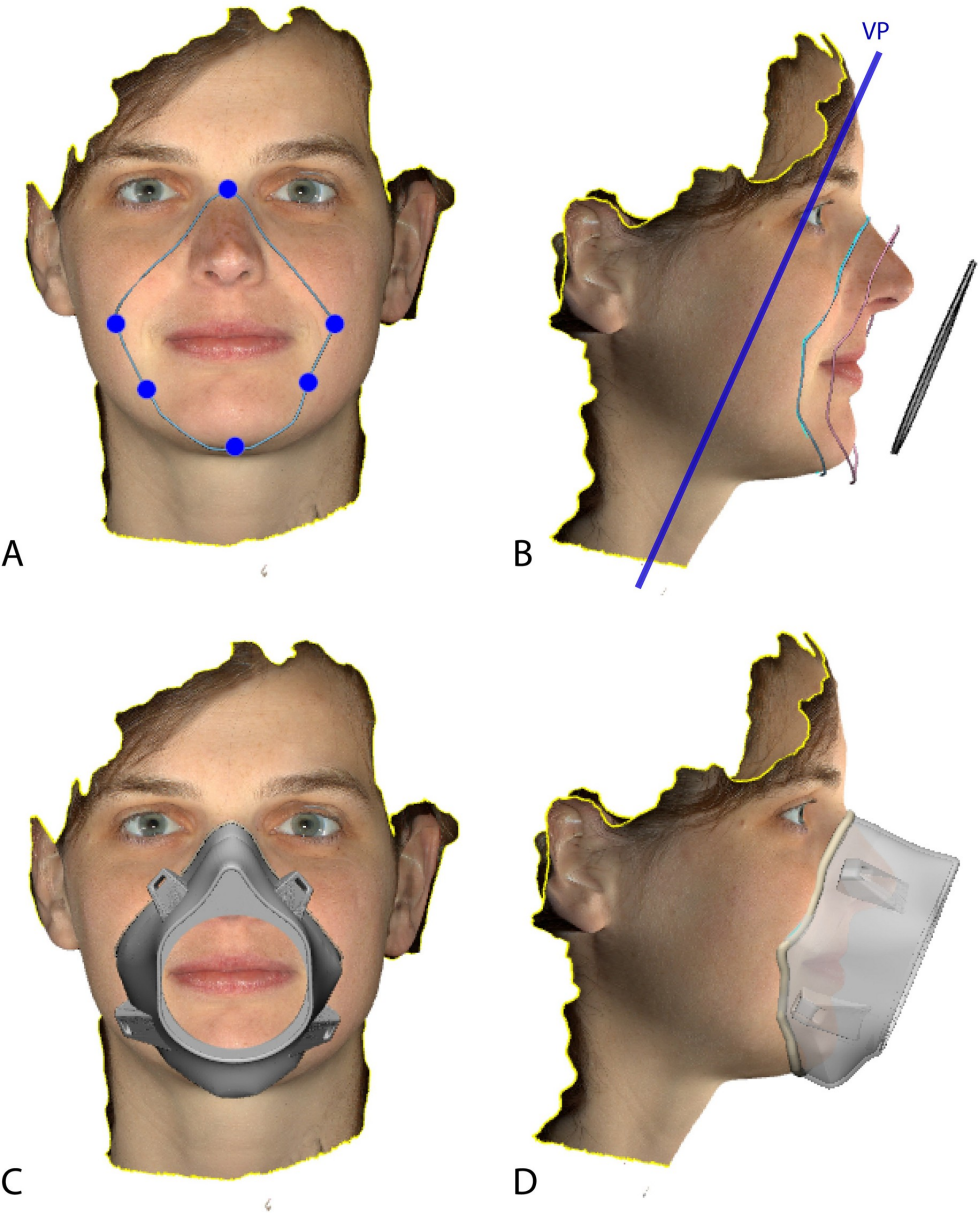
A. Base curve built based on the 6 control points (C1 to C6) of the subject’s face. B. Base curve attached to the surface, then the translated copy and the base filter. C. Frontal view of the face specific mask after the loft function. D. Side view of the face specific mask shown in transparent.

**Step 2: Control points and planes calculations.** The nine landmarks and the OBJ face file are imported into 3-matic software (Materialise, Leuven, Belgium). In this step, the landmarks are used to automatically define a number of planes and control points, which serve as a base for the next step. The details of these control points and planes are further described in Table 1 and Figs 1B and 2B with three main planes: MidFace (MF), Vertical plane (VP) and Horizontal plane (HP).

**Step 3: Base components construction.** Two base components are required to build the main body of the mask: contour curve and base of the filter. The six indicated control points in Table 1 are used to build a contour curve. This curve is the base of the mask and guarantees a tight fit on the face because it is restricted to be attached to the face surface (Fig 2A).

The base of the filter was obtained from online open source public library as Standard Tessellation Language (STL) file [10]. It was imported into the project then relocated on the plane parallel to the vertical plane (VP) and going through a point translated anteriorly 20 mm from the nose tip (Ntp).

**Step 4: Mask building.** To allow for additional space at the nasal dorsum a copy of the base contour curve is translated anteriorly half of the distance between Ra and Ntp (Fig 2B). Then the loft function is used to build the surfaces from base curve to translated curve and from translated curve to filter base. A thickness of 2mm is assigned to the mask body. Next, a soft circular rim is designed by subtraction of the mask body from a circular loft applied to the base curve. This rim adjustment will facilitate the wearing comfort (Fig 2C and 2D).

**Fig 3:**
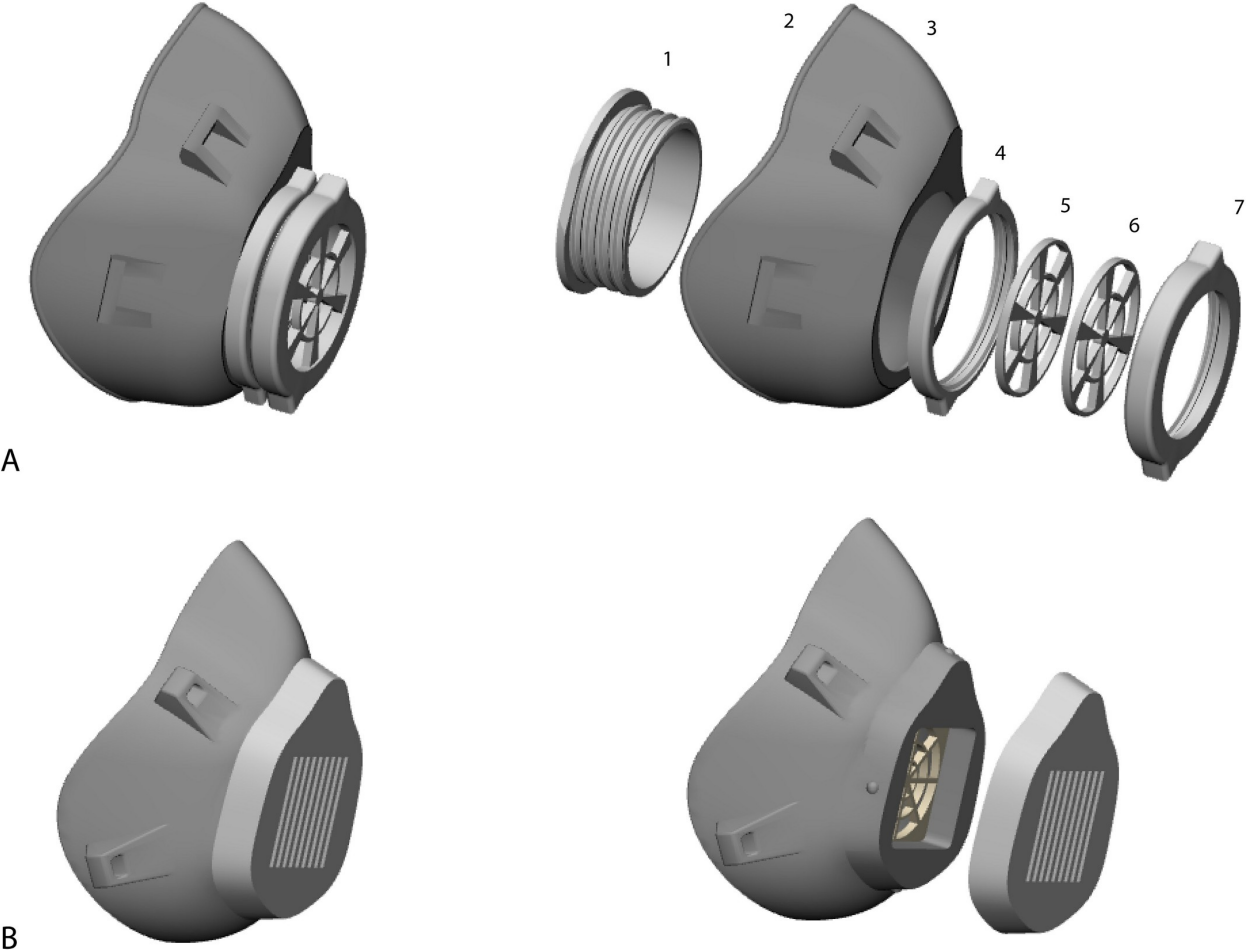
A. The screw drive mask assembled at the left and in separate components on the right. B. A universal mask design with alternative locking filter system (click cover).

**Fig 4:**
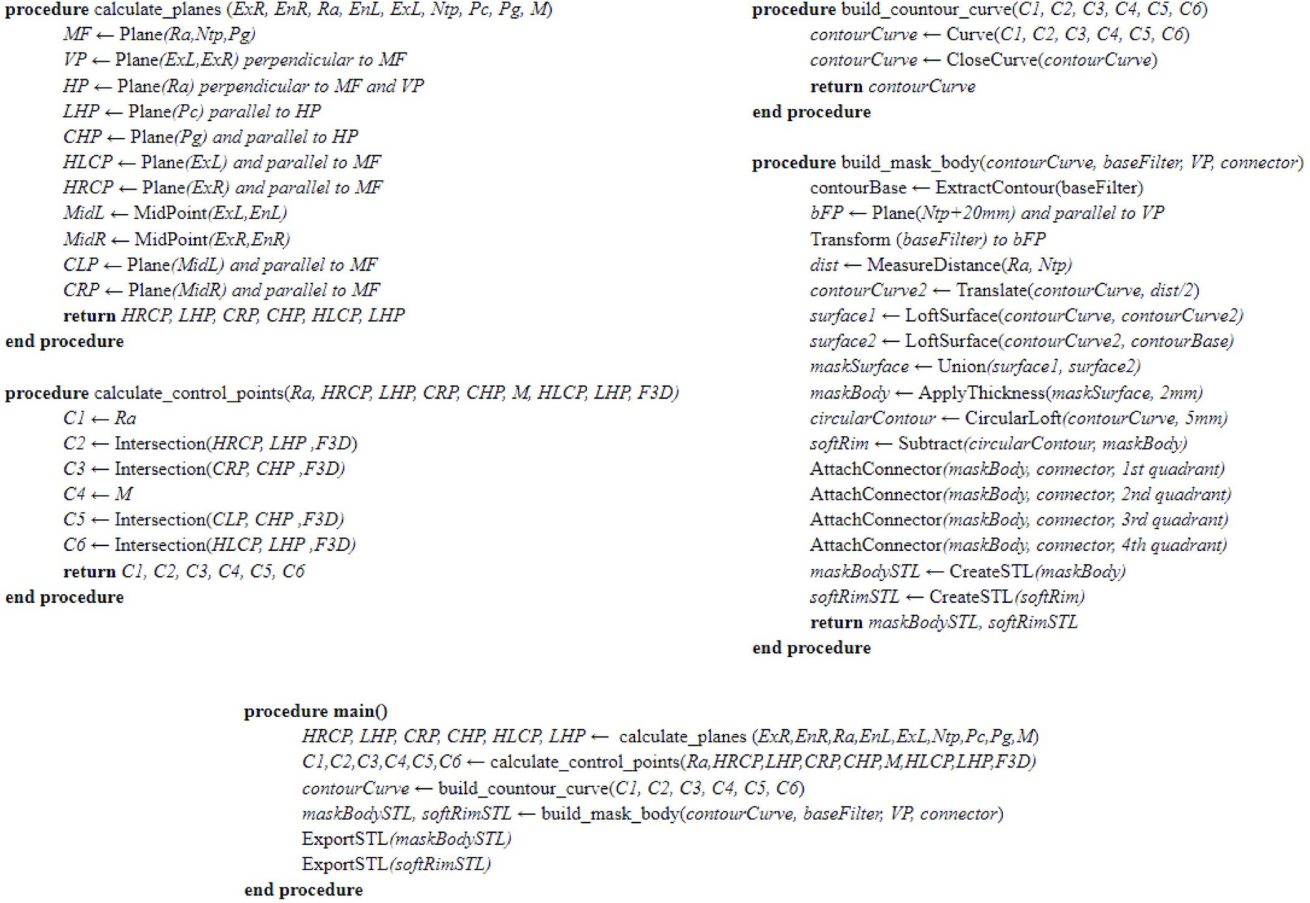
The pseudocode describing the proposed algorithm. Variables have been identified in the text and Table 1.

**Step 5: Mask completion.** Four connection parts are attached to the mask body as shown in Fig 3A. The mask can be personalized by adding a label containing an identification number of the user. The mask is then exported as STL file format to be printed. The soft circular rim is also exported as a separate STL file. Fig 4 presents the pseudocode of the proposed design algorithm from step 2 to step 5 where all these steps are fully automatic without user interference. it has to be noted that some values could be adjusted according to testing. The main variables have been identified in the text.

### Mask 3D printing and assembly

Seven parts are printed as shown in Fig 3A: 1. Washer; 2. Soft rim; 3. Mask body; 4. Grating; 5. Inner filter cover; 6. Outer filter cover; 7. Female. All components were printed using Objet Connex 350 (Stratasys, Eden Prairie, MN USA) a polyjet 3D printer with layer thickness of 30μm in hard transparent material (VeroClear) and only the soft rim was printed in a rubber-like soft transparent material (Tango+). This material is best sterilized using gas plasma as described by Shaheen et al. [11].

This screw drive locking system is assembled in the same order as presented in Fig 3A. First the washer is inserted into the mask opening and locks in. Then the grating is screwed on the washer to fixate the washer to the mask. The inner cover is inserted in the washer followed by the filter and the outer cover. Finally the female part is screwed on the washer to close the system.

Three masks were printed for the testing subject and subjective evaluation was conducted: 1. Face specific mask following the proposed design. 2. Universal mask design with screw drive locking system [12]. 3. Universal mask design with click locking system (Fig 3B).

## Results

### Face specific vs universal mask design

**Fig 5:**
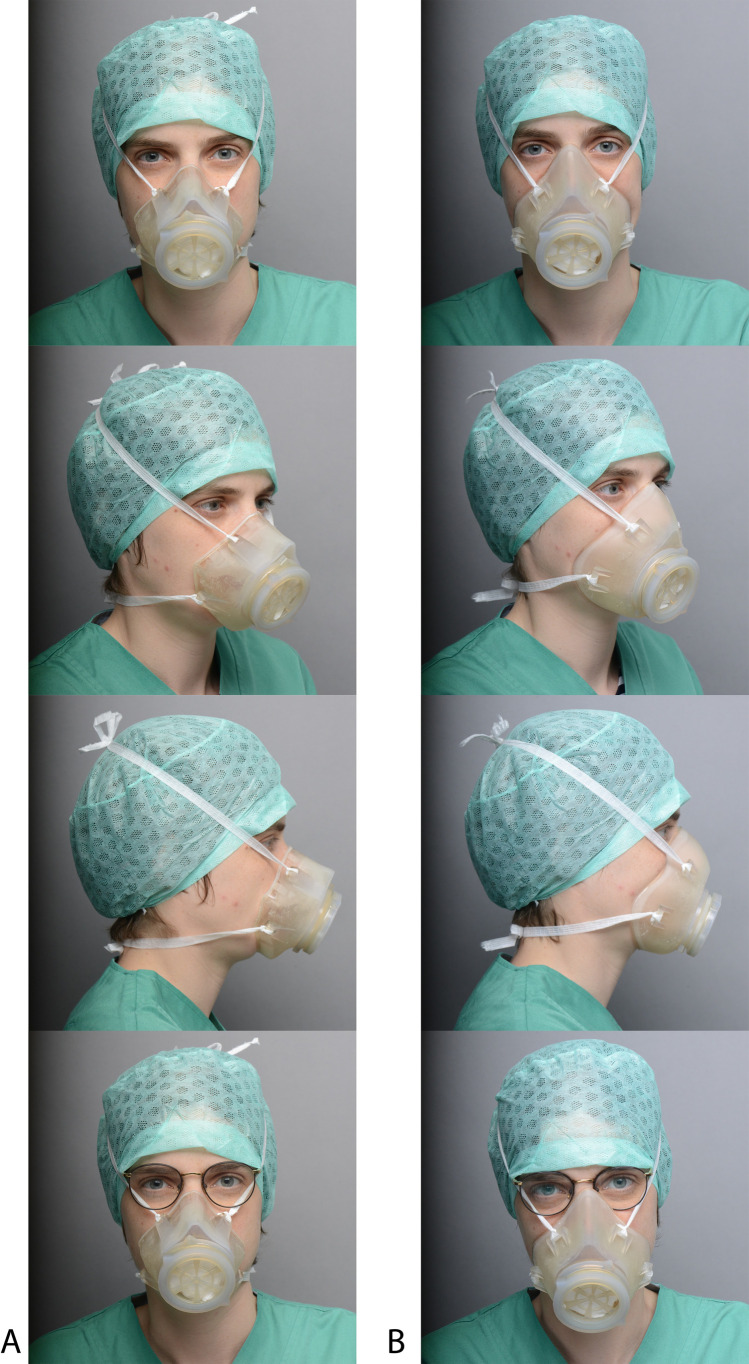
A. Different views of the test subject trying on the face specific 3D printed mask vs B. Different views of the test subject trying on the universal mask design.

The face specific mask was perfectly adjusted to the facial contours (Fig 5A). The soft circular rim not only provided additional comfort, but also contributed to an improved closure of the mask since it was built based on the contour curve. There were no problems when combining the mask with glasses and there was a clear field of view.

Carrying the universal mask (Fig 5B), the volunteer expressed discomfort at the nasal dorsum and cheeks. The base of the mask could not fit properly at the chin and nose region simultaneously, causing a gap between mask and skin. The upper part of the mask partially blocked the field of view and prohibited convenient wearing of glasses.

### Click vs screw drive filter locking system

The mask with a click locking system to contain the filter was fragile and broke when it was disassembled to change the filter (Fig 3B). Therefore, we adopted the design of screw drive locking system (Fig 3A), which is straightforward to assemble, strong for multiple usages hence expanding the lifespan. Additionally, this filter design stands independent from the mask body, which allows the reprinting of any part in case it is damaged.

## Discussion

In this paper we have presented an automatic approach to design a 3D face specific mask. Automation of the mask design according to the individual facial features allows for mass production with guaranteeing optimal protection and comfort. Using a universal design facilitates rapid manufacturing but fails to completely seal the nose and mouth. Besides decreasing the comfort, also the position of glasses can be hindered when the design is not adjusted. On the other hand, fully manual designing of the mask is laborious and time consuming, constraining the swift implementation during a crisis situation.

According to the subjective evaluation, the 3D printed face specific mask was reported to be more comfortable, and felt to create an efficient closure of the nose and mouth. Wearing eye protection is essential to prevent droplet contamination; therefore it is important that a facemask can be easily combined with glasses. Further objective mask fit tests are needed and are part of an ongoing research. Screw drive locking system of the filter is easily integrated in the mask design and facilitates replacement of a single part when damage may occur.

The mask creation as proposed in this paper can be completely automatic. Some values were chosen to guarantee as minimal as possible user interference. However, it is recommended to allow some degree of designer interaction with the possibility for manual adjustments. The proposed method permits the designer to relocate the control points and adjust the base contour to meet special requirements, e.g. to avoid skin lesions. It is possible to select different control points or create alternative reference planes to adapt the design according to population specific characteristics. Therefore, this method should be regarded as a guideline strategy to make fully automatic design possible, while maintaining sufficient designer interaction.

It can be argued that this implementation was based on commercial tools (software packages and imaging camera). However, other open source software packages could implement the same approach following the provided pseudocode. The authors chose pseudocode because it is environment-independent and facilitates programming to other developers independent from the language used. First, the anatomical landmarks were automatically provided from the Vectra software, but this could be solved by implementing the algorithm suggested by Codari et al. for computer aided cephalometric landmark annotation for 3D data [13]. Moreover, this step is not time consuming since only nine landmarks are needed and it can be manually placed. The accuracy of placing cephalometric landmarks is in the error range of 2mm which is not significant for the mask design [14]. Second, with the rise of scripting using Python, a number of open source software, such as Blender (Blender Foundation, Amsterdam, the Netherlands), offer the possibility of scripting to facilitate and automate the design process. Third, 3D stereophotogrammetry is currently widely used with different medical treatments such as maxillofacial surgery, orthodontic treatments, plastic surgery, etc. This 3D face scan can also be realized using 3D laser scanning which is incorporated in a number of Cone Beam Computed Tomography systems [9]. Furthermore, using high tech smartphones and specific apps, a 3D photo can be captured as described by Swennen et al. [15].

Even though the main focus of this work was the design, a fully functioning mask doesn’t rely solely on the design even if it plays a major role. Other factors should be properly investigated before using these 3D masks in practice such as the toxicity of the printed material. In this paper we used the polyjet technology that provides rubber-like and hard materials, which could be simultaneously printed. However, we chose to print the components separately before assembling the complete mask in order to facilitate the procedure with the use of 3D printers that can only print a single material at once. Alternatively to our proposed procedure, one could also only print the hard components and replace the soft rim with commercially available soft edge tape. Whether the printed mask is suitable for continuous and comfortable breathing needs to be clarified. Therefore, the authors plan to explore other 3D printers that could be used to construct farm labs with the following properties: 1. Sufficient printing tray size conform the required manufacturing capacity. 2. Printing materials that are considered suitable and non-toxic. 3. Preferably the capability to print soft and hard materials, not necessarily at the same time. 4. Budget friendly with accuracy at least 120μm [16]. Another important factor is the filter, we recommend the use of disposable filters that are conform quality FFP2 (N95) and FFP3 (N99). The proposed design can be adapted to any shape or size of the filters used and is able to contain different kinds of filters. In an ongoing study, profound testing will be performed according to the European Standard (“Respiratory protective devices—Filtering half masks to protect against particles—Requirements, testing, marking”—EN 149+A1 (2009)) and to the European specific performance tests for reusable masks with separate filters (EN 1827). These investigations should reveal which filters are suitable and comply with the EN 149 standards. Moreover, the tight closure of the mask will be evaluated in accordance with the requirements concerning leakage. The specific tests are described in the guidelines EN 149+A1.

For optimal safety, the 3D printed mask should be sterilized. To guarantee a prolonged lifespan of the mask, it is important to test the deformation of the material following sterilization. Steam heat and gas plasma were previously explored for this polyjet 3D printer and deformations were found after steam heat sterilization [11]. Once a 3D printer passes the above-mentioned tests, different sterilization techniques will be tested to reach the best sterilization method for the specific 3D printing material.

## Conclusion

Automatic and face specific manufacturing of facemasks is feasible through the CAD-CAM technology that provides comfortable and durable alternative to regular masks. Implementation in clinical practice and daily use requires additional research on the most suitable printing material, sterilization technique and compliance with international regulations.
